# Public Health Messaging for Wildfire Smoke: Cast a Wide Net

**DOI:** 10.3389/fpubh.2022.773428

**Published:** 2022-04-27

**Authors:** Erin M. Shellington, Phuong D. M. Nguyen, Karen Rideout, Prabjit Barn, Anna Lewis, Margaret Baillie, Sue Lutz, Ryan W. Allen, Jiayun Yao, Christopher Carlsten, Sarah B. Henderson

**Affiliations:** ^1^Legacy for Airway Health, Vancouver Coastal Health Research Institute, Vancouver, BC, Canada; ^2^Environmental Health Services, British Columbia Centre for Disease Control, Vancouver, BC, Canada; ^3^Port Alberni Air Quality Council, Port Alberni, BC, Canada; ^4^Community Stakeholder Committee, Legacy for Airway Health, Vancouver Coastal Health Research Institute, Vancouver, BC, Canada; ^5^Faculty of Health Sciences, Simon Fraser University, Burnaby, BC, Canada; ^6^British Columbia Observatory for Population and Public Health, British Columbia Centre for Disease Control, Vancouver, BC, Canada; ^7^Division of Respiratory Medicine, Department of Medicine, Faculty of Medicine, University of British Columbia, Vancouver, BC, Canada; ^8^Faculty of Medicine, School of Population and Public Health, University of British Columbia, Vancouver, BC, Canada

**Keywords:** wildfire, air pollution, air quality, public health, communication, knowledge translation

## Abstract

Wildfire smoke events are increasing in British Columbia (BC), Canada and environmental and public health agencies are responsible for communicating the health-related risks and mitigation strategies. To evaluate and identify opportunities for improving public communications about wildfire smoke and associated health risks we collaborated with end-users and developed a 32-question online survey. The survey was deployed province-wide from 29 September to 31 December 2020 following a severe wildfire smoke episode, which impacted large parts of BC. Using a convenience sample, we disseminated the survey through email lists, radio advertisements, a provincial research platform, and snowball methods. There were 757 respondents, who were generally representative of provincial demographics. Respondents indicated that they receive wildfire smoke messages from diverse sources, including: websites, social media, radio, and television. Radio was identified as the most important source of information for populations that may have increased exposure or health risks, including Indigenous respondents and those working in the trades. Respondents with lower educational attainment expressed that messaging should be simplified. Environmental and public health agencies should continue to share wildfire smoke messages using diverse methods, ideally tailoring the messages and methods to specific populations at risk for exposure and health effects.

## Introduction

British Columbia (BC), Canada has experienced increasingly severe wildfire seasons over the past decade (1, 2). Exposure to wildfire smoke can have negative impacts on human health, especially for people living with lung or cardiovascular disease, older adults, pregnant women, infants and children, and marginalized populations (3). Given that wildfire smoke exposures will continue to increase in frequency and severity as the global climate changes, air quality alerts and public health messages related to wildfire smoke are of growing importance to protect public health (4).

Evaluation of wildfire smoke communications is limited. In one of the first studies conducted, more than 80% of participants in Humboldt County, California reported hearing at least one public service announcement about smoke (5). A recent systematic review found that social media is an important source of information during smoke events and compliance is higher when messages are simpler (6). Twenty-four household interviews conducted during a wildfire emergency in Australia found that smoke exposure messages were not timely or practical enough, and that they competed with fire risk messages (7). Analyses of SmokeSense (a mobile phone application) user data in the US found that message uptake depended more on individual receptiveness than on demographic factors (8). Lastly, a case report from the 2007 San Diego fires revealed that non-English speaking communities, including migrant and undocumented individuals are less likely to receive or understand messages; moreover, fear of deportation was considered more risky than the fires or smoke (9).

**Figure 1:**
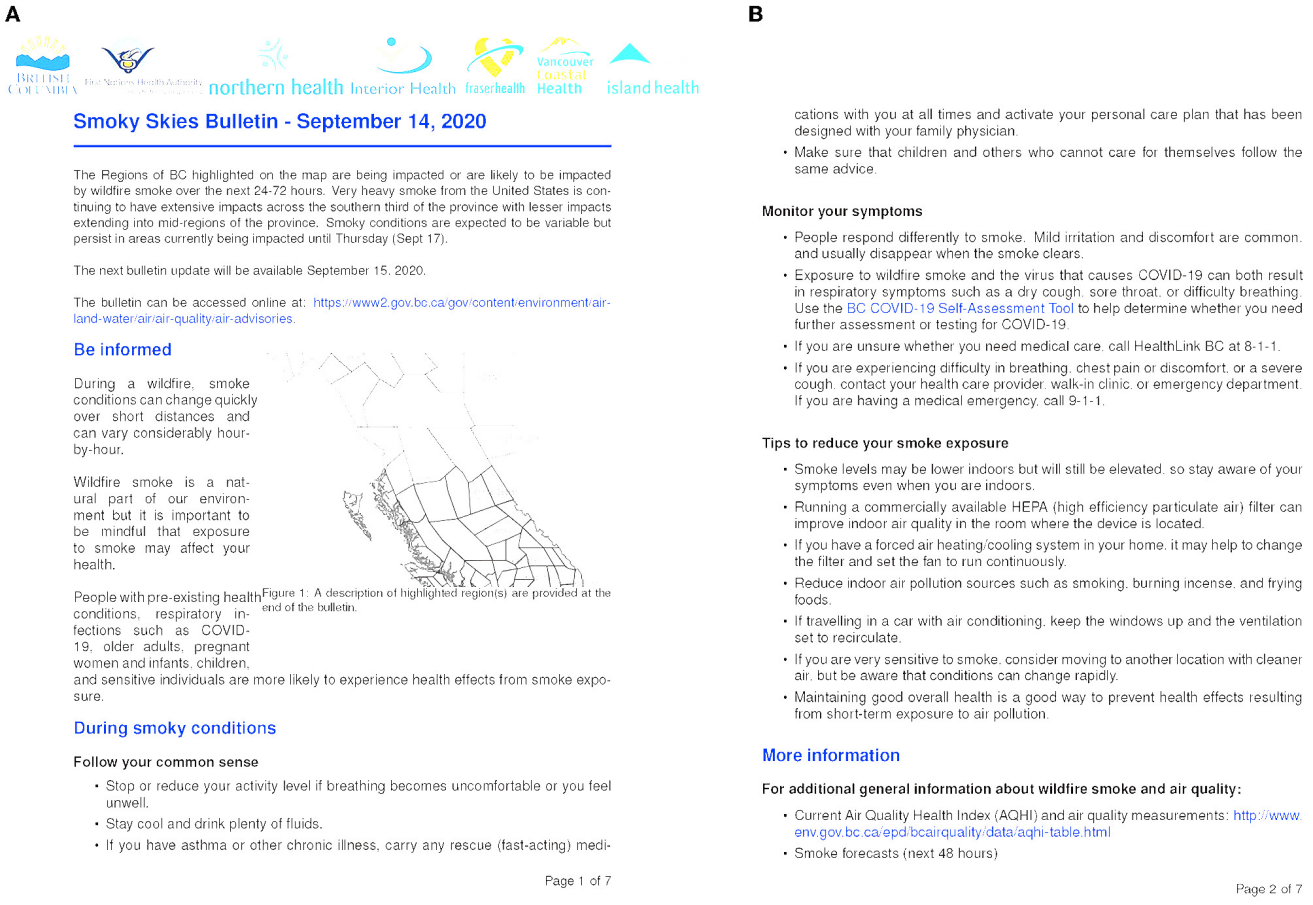
Smoky Skies Bulletin from September 2020 with **(A)** air quality information and **(B)** health information.

In BC, wildfire smoke response involves regional, provincial, and federal agencies that have environmental and public health responsibilities. Regional and provincial environmental agencies issue smoky skies bulletins and air quality advisories using observations and smoke forecasts from provincial and national partners. These messages include details on the location of the smoke, expected duration, and severity as well as how to protect oneself from the negative health effects of wildfire smoke exposure (Figure 1). The national Air Quality and Health Index (AQHI) has also been adapted for better performance during wildfire smoke episodes in BC, and these AQHI values are made available through multiple websites and smartphone applications (10). The AQHI is a rating (1–10+) based on a combination of pollutants and it provides health protection information for the general public and at-risk populations. Regional health authorities and the BC Centre for Disease Control provide more detailed health information through various methods, including newly translated fact sheets for lay audiences (11). These agencies use conventional platforms and social media to communicate different aspects of wildfire smoke, air quality, health effects, and health protection to the public.

To date, there has been no evaluation of wildfire smoke communications to assess how people in BC receive, understand, and implement advice to reduce exposure to wildfire smoke. This study aims to provide such evaluation and identify opportunities to improve future communications. A co-development process with government agencies and community and patient partners was used to maximize the relevance of the findings.

## Methods

We engaged patient and community partners to co-develop a survey about wildfire smoke messaging for the public, and refined it with input from key environmental and public health agencies (April–July 2020; Supplementary Material A). Patient partners are individuals with lived experience of COPD and asthma (authors MB and SL) and the community partner works for a local air quality council (author AL). The survey was hosted on the Qualtrics tool (www.ubc.qualtrics.com) and was preceded by a cover letter for informed consent. It comprised of 32 voluntary closed-ended questions, which collected information on demographics and sought to evaluate:

- How people receive wildfire smoke information and public health messages.
- Whether people understand the messages.
- Whether people use any of the information to reduce their smoke exposure.

The 2020 BC wildfire season was below average with respect to the number of fires and the area burned, but the survey was distributed in September following a prolonged and widespread wildfire smoke episode caused by long-range transport of smoke from fires in California, Oregon, and Washington states (12). The survey closed on 31 December 2020.

The province of BC has a population of 5 million people, and a landmass of almost 1 million km^2^, with 64% covered by forest. According to the 2016 census, approximately 50% of the population lives in greater Vancouver, 28% identify as immigrants, and 6% identify as Indigenous.

We recruited a convenience sample of survey respondents through government, health, and organization social media posts (e.g., https://twitter.com/BCLungAssoc/status/1317232503959085056) email distribution lists through community networks; personal networks; community newsletters; paid radio advertisements; and a provincial research study participant recruitment platform (www.reachbc.ca/). Participants had the option to enter a random draw to win one of seven gift cards valued at $25-$100. All data were analyzed using frequency tables and visualized with a Shiny R web application (Supplementary Material B: https://ehs-bccdc.shinyapps.io/2020smoke_survey/).

## Results

**Figure 2:**
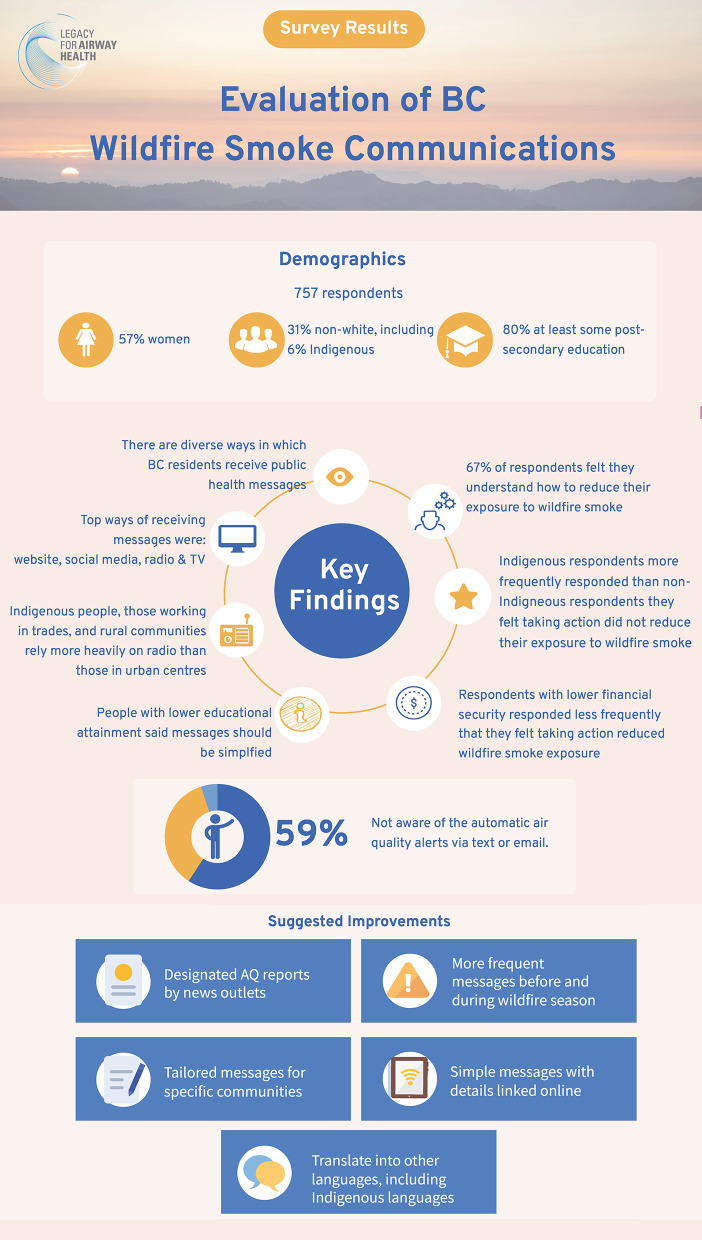
Summary of survey results of wildfire smoke communications in British Columbia.

There were 757 respondents. Fifty-eight percent identified as women, 31% identified as non-white (including 6% Indigenous), 52% lived in greater Vancouver, and over 80% had at least some post-secondary education (Figure 2).

**Figure 3:**
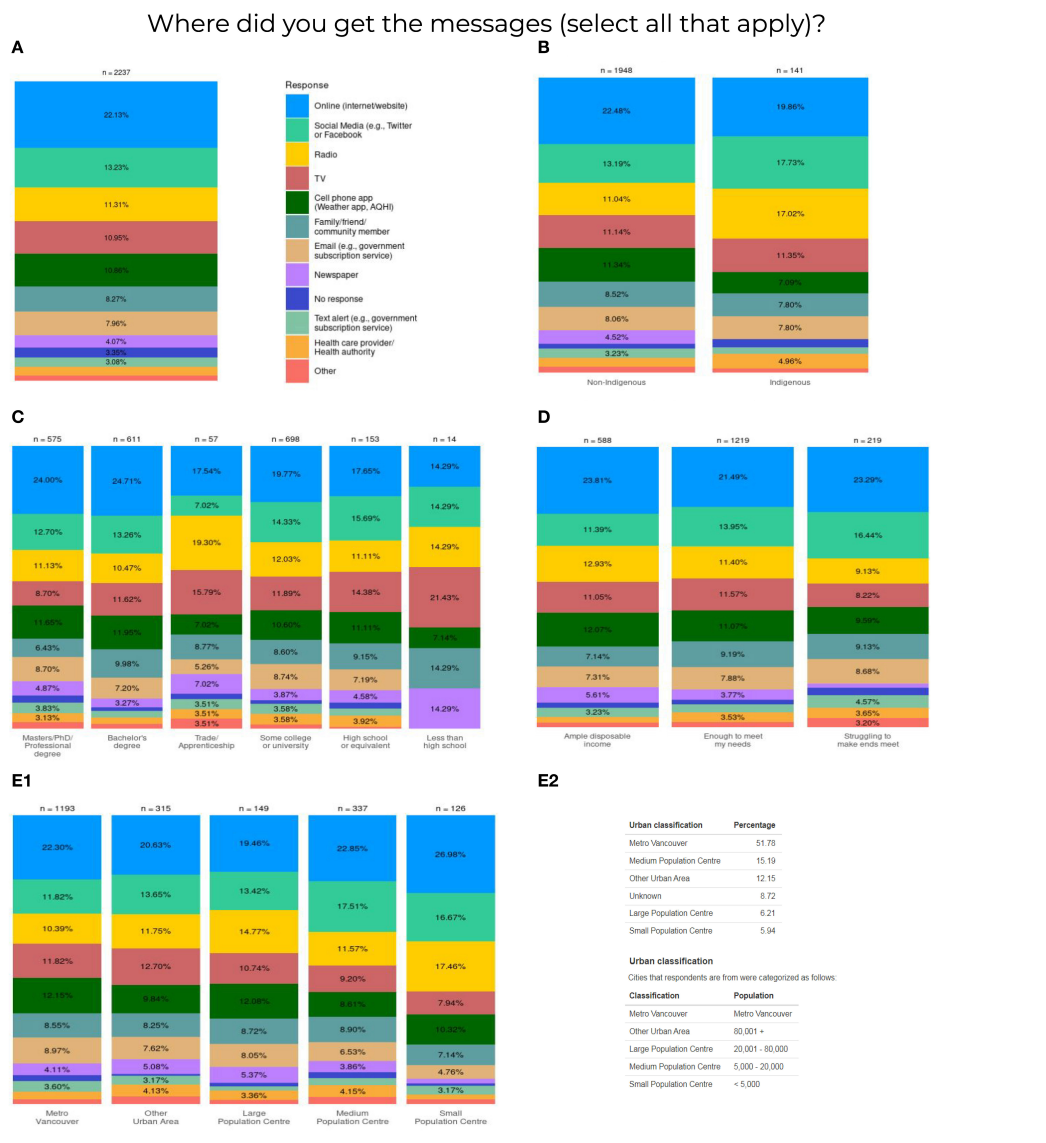
**(A)** Aggregate and subgroup [**(B)** ethnicity, **(C)** educational attainment, **(D)** financial security, **(E1)** urban status with **(E2)** urban classifications] data representation on where survey respondents get information related to wildfire smoke communications.

The results indicate that people in BC access wildfire smoke information in diverse ways. The top categories included: (1) websites; (2) social media; (3) radio; and (4) television (Supplementary Material B, Part 1, Q3). Sub-group frequencies showed that radio may be an important source of information for respondents who identify as Indigenous, work in the trades, or live in small population centers (i.e., <5,000 population; Figure 3). Websites were the most frequent way to access information for non-Indigenous respondents, those with high educational attainment and those in small population centers. However, nearly 60% of respondents were unaware of a new automatic text/email service for air quality bulletins and advisories provided by the provincial government (https://www2.gov.bc.ca/gov/content/environment/air-land-water/air/air-quality/air-advisories/air-quality-subscription-service) and Metro Vancouver Regional District (http://www.metrovancouver.org/services/air-quality/engagement/mailing-list/Pages/default.aspx) (Supplementary Material B, Part 1, Q6).

Over 67% of respondents indicated that they understand the information provided by public health messages on how to reduce their exposure to wildfire smoke (Supplementary Material B, Part 2, Q5). This did not vary by rurality or ethnicity but did vary by education. Those with lower educational attainment indicated a poorer understanding of exposure reduction strategies. Over 88% of respondents indicated they followed recommendations to reduce exposure (Supplementary Material B, Part 3, Q4). However, over 18% of Indigenous respondents who took recommended actions felt unsure about whether their exposure was reduced compared with 11% of non-Indigenous respondents (Supplementary Material B, Part 3, Q8).

Respondents suggested that wildfire smoke advisories could be improved through: designated air quality reports by news outlets; tailored messages for specific communities; and more frequent messaging before and during the wildfire season (Supplementary Material B, Part 1, Q7). Respondents also suggested that messages should be simple, but with additional details available online. Nearly 27% of respondents with less than a high school education indicated that messages should be simpler and easier to understand. Respondents identified many ways to increase the reach of messages, including translation into languages spoken by Indigenous and immigrant populations in BC.

## Discussion

The survey provides useful insights into the diverse ways that wildfire smoke messages are received by the public in BC. The survey was distributed during an episode of widespread wildfire smoke in the absence of proximate wildfires. This provided a rare opportunity to evaluate wildfire smoke communications that were not conflated with wildfire risk communications. The results demonstrate the importance of using different platforms, and highlight the importance of radio communications for some populations, including Indigenous peoples, certain occupational groups, and rural areas. Radio may have greater reach in many parts of BC with limited cellular connection and/or high-speed internet. Additionally, hand (or two-way) radios may be a useful communication tool for isolated communities. In non-urban areas, particularly in interior BC, wildfire smoke is more frequent and severe due to the local climate and weather patterns (e.g., desert and thunderstorms). The landscape and topography in interior BC also contributes to the accumulation of smoke in valleys, changing winds, and limited cellular service. Because wildfire smoke is increasing in frequency and duration, message fatigue and waning perceived risk may further complicate wildfire smoke communications. Using diverse modes of communication, including radio, should be prioritized for wildfire smoke communications in the province.

Most communications related to wildfire smoke information are in English, and the survey was only administered in English. As such, we have not captured the perspective of people who do not read or speak English, which could include Indigenous and immigrant communities. This language gap in wildfire communications was highlighted as a barrier among non-English speaking communities in Southern California (9). In BC, migrant farm workers are often subject to poor working and living conditions, live and work in or near areas frequently affected by wildfire smoke, and have limited social supports and connections (13). Thus, they are at high-risk of being exposed to wildfire smoke with little to no means to protect themselves. The limited distribution and/or awareness of cultural and linguistically tailored wildfire smoke risk communication makes migrant farm workers particularly vulnerable.

Respondents, particularly those with lower educational attainment, indicated that message content should be simplified. This could achieved by lowering the reading level and using graphics to minimize language and literacy barriers. Messages for higher-risk populations could be amplified using social media or by reaching out to local media outlets, farm associations, and community representatives. The messages should aim to reach those with limited access to communications and emphasize groups at high-risk of smoke exposure.

This study adds to the limited literature evaluating wildfire smoke communications and is broadly consistent with findings of prior studies. Our results provide information for BC agencies about effective communication methods and opportunities for improvement. The survey respondents were geographically and ethnically representative of the BC population, though they were skewed toward women and those with post-secondary education. Through the co-developing the survey, we are able to share the results with many stakeholders who can influence future wildfire smoke communications in BC.

## Conclusions

People in BC receive messages about wildfire smoke, air quality, health effects, and health protection via different modes of communication. Local radio is particularly important for Indigenous people, rural communities, and those working in the trades. Effective communication about reducing smoke exposure may help to reduce the burden of disease attributable to wildfire smoke as the climate changes.

## Data Availability Statement

The original contributions presented in the study are included in the article/Supplementary Material, further inquiries can be directed to the corresponding author/s.

## Ethics Statement

The studies involving human participants were reviewed and approved by University of British Columbia Behavioural Research Ethics Board. Written informed consent for participation was not required for this study in accordance with the national legislation and the institutional requirements.

## Author Contributions

Material preparation, data collection, and analysis were performed by ES, PN, CC, and SH. The first draft of the manuscript was written by ES, CC, and SH. All authors contributed to the study conception and design, commented on previous versions of the manuscript, read, and approved the final manuscript.

## Funding

This study was funded by Legacy for Airway Health, a donor-funded research program for the paid radio advertisements, patient partner honoraria, and draw prizes.

## Conflict of Interest

The authors declare that the research was conducted in the absence of any commercial or financial relationships that could be construed as a potential conflict of interest.

## Publisher's Note

All claims expressed in this article are solely those of the authors and do not necessarily represent those of their affiliated organizations, or those of the publisher, the editors and the reviewers. Any product that may be evaluated in this article, or claim that may be made by its manufacturer, is not guaranteed or endorsed by the publisher.

## Supplementary Material

Supplementary Material A Wildfire smoke communication survey questions.

Supplementary Material B Wildfire smoke communication survey results web app - https://ehs-bccdc.shinyapps.io/2020smoke_survey/.
