# Supplementary material for: Public Health Messaging for Wildfire Smoke: Cast a Wide Net
Source: Front Public Health. 2022 Apr 27;10:773428. doi: 10.3389/fpubh.2022.773428 (PMC9132092; doi:10.3389/fpubh.2022.773428)
Supplement: Supplementary Material A — Wildfire smoke communication survey questions. [file Data_Sheet_1.pdf]

Wildfire Smoke Exposure and Public Health Messages

The first block of questions is designed to evaluate how people are receiving the wildfire smoke advisories and public health messages.

An example of a message about wildfire smoke exposure and public health might be:

BRITISH COLUMBIA | First Nations Health Authority, Health through wellness | northern health | Interior Health | fraserhealth | Vancouver Coastal Health | island health

Smoky Skies Bulletin - September 14, 2020

The Regions of BC highlighted on the map are being impacted or are likely to be impacted by wildfire smoke over the next 24-72 hours. Very heavy smoke from the United States is continuing to have extensive impacts across the southern third of the province with lesser impacts extending into mid-regions of the province. Smoky conditions are expected to be variable but persist in areas currently being impacted until Thursday (Sept 17).

The next bulletin update will be available September 15, 2020.

The bulletin can be accessed online at: https://www2.gov.bc.ca/gov/content/environment/air-land-water/air/air-quality/air-advisories.

Be informed

During a wildfire, smoke conditions can change quickly over short distances and can vary considerably hour-by-hour.

Wildfire smoke is a natural part of our environment but it is important to be mindful that exposure to smoke may affect your health.

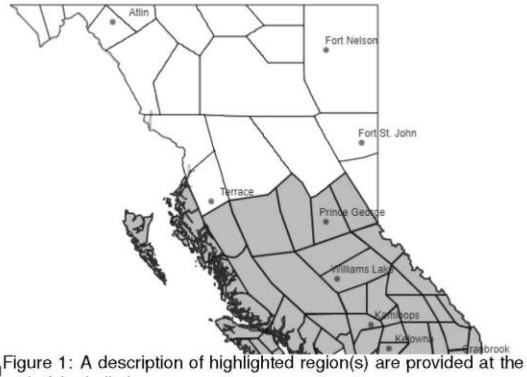

Figure 1: A description of highlighted region(s) are provided at the end of the bulletin.

People with pre-existing health conditions, respiratory infections such as COVID-19, older adults, pregnant women and infants, children, and sensitive individuals are more likely to experience health effects from smoke exposure.

During smoky conditions

Follow your common sense

- Stop or reduce your activity level if breathing becomes uncomfortable or you feel unwell.
- Stay cool and drink plenty of fluids.
- If you have asthma or other chronic illness, carry any rescue (fast-acting) medi-

Page 1 of 7

Were you aware of any wildfire smoke messages for your community during this past (2020) summer or previous wildfire seasons, such as 2015, 2017 or 2018?

- ○ Yes
- ○ No

Under what circumstances have you ever heard or seen any of these types of messages about wildfire smoke in your community (check any that apply)?

- ☐ Information only (e.g., reports of a local wildfire)
- ☐ Advisory, Warning, or Alert (e.g., smoky skies bulletin on previous page)
- ☐ Evacuation (due to smoke from fire and ***not*** due to physical danger from a fire)
- ☐ I have not heard or seen any messages about wildfire smoke before
- ☐ Not sure

Where did you get these messages (select any that apply)?

- ☐ TV
- ☐ Newspaper
- ☐ Radio
- ☐ Online (internet/website)
- ☐ Social Media (e.g., Twitter or Facebook)
- ☐ Cell phone app (Weather app, AQHI)
- ☐ Email (e.g., government subscription service)
- ☐ Text alert (e.g., government subscription service)

- [ ] Family/friend/community member
- [ ] Health care provider/ Health authority
- [ ] Other ____

Do you recall experiencing (e.g., seeing or feeling the effects of) wildfire smoke in your community when a Smoky Skies bulletin was issued?

- ○ Yes
- ○ No
- ○ Unsure

Do you feel that the messages about wildfire smoke in your community gave you enough time to prepare for the smoke?

- ○ Yes
- ○ No
- ○ Did not receive an alert/warning for my area

Do you know that you can sign up to automatically receive air quality messages (Smoky Skies Bulletins and Air Quality Advisories) via your email address and/or mobile phone number? *https://aqss.nrs.gov.bc.ca/subscription.html*

- ○ Yes
- ○ No

In what ways could these public health messages about wildfire smoke be improved (Please select your top 3 options)?

- [ ] Make messages available in more languages (Please provide example of a language) ____
- [ ] Make messages simpler and easier to understand
- [ ] Provide simple messages with more details through a link

- [ ] Share messages more widely (Provide example) ____

- [ ] Share messages more frequently throughout the wildfire season

- [ ] Share messages **before** the wildfire season

- [ ] Tailor messages to specific communities

- [ ] All news reports (tv, online, newspaper, etc.) should have a section about wildfire smoke during wildfire season

- [ ] Other ____

Has the current COVID-19 pandemic changed your awareness about local air quality?

○ Yes

○ No

○ Unsure

Do you believe that exposure to wildfire smoke could affect your likelihood of being infected with COVID-19?

○ Yes

○ No

○ Unsure

The second block of questions is designed to evaluate whether people understand the messages related to wildfire smoke exposure.

How concerned are you about how wildfire smoke affects your health, a family member's health, or a close friend's health?

○ Not at all concerned

○ Somewhat concerned

○ Very concerned

How well do you feel you understand the ***information*** related to:

	None at all – minimal	A little bit - some	Most of the information makes sense to me	I have not seen information about this before
Predicted or actual wildfire smoke levels in your community, such as an advisory or alert?	○	○	○	○
The health impacts of wildfire smoke?	○	○	○	○
Who might be most susceptible to wildfire smoke?	○	○	○	○
Ways to reduce your exposure to wildfire smoke?	○	○	○	○

The third block of questions is to evaluate whether people use any of the public health advice to reduce their smoke exposure.

Do you know what measures or actions are advised when there is wildfire smoke in your community?

○ Yes

○ No

Where do you typically get information related to reducing your exposure to wildfire smoke?

○ Government sources,such as twitter accounts, government websites or email or text alerts.

○ Media sources, such as tv, radio, media websites

○ Other (please provide example)

____

Have you heard of the following ways to respond to wildfire smoke exposure (check all that apply)?

- ☐ Use a portable air filter, air purifier, high efficiency or HEPA filter air cleaner at home
- ☐ Seek a clean air refuge in your community (a large, air-conditioned indoor space such as a public library or shopping mall)
- ☐ Stay indoors with windows and doors closed, while avoiding the heat from hot weather
- ☐ Limit outdoor activities
- ☐ Use a face mask, such as an N95 respirator or covering over your nose and mouth
- ☐ Follow your action plan (use of your medication as prescribed by your health care provider)
- ☐ Cancel or postpone outdoor activities
- ☐ Other ____
- ☐ I have not heard of any ways to respond to wildfire smoke exposure

Did you follow any of the recommendations above?

- ○ Yes
- ○ No

Did you share any of the recommendations with friends/family/community members that might be affected?

- ○ Yes
- ○ No

Which action(s) did you or others you know take (Please check any that apply)?

☐ Used a portable air filter, air purifier, high efficiency or HEPA cleaner at home

☐ Went to clean air refuge in the community, including a public library, shopping mall, or other large air-conditioned space

☐ Stayed indoors with windows and doors closed, while avoiding heat from hot weather

☐ Limited outdoor activities

☐ Used a mask

☐ Followed your action plan (used of your medication as prescribed by your health care provider)

☐ Canceled or postponed outdoor activities

☐ Other ____

What prevented you from taking actions? (check any that apply)?

☐ I did not need to; I wasn’t affected or concerned

☐ I did not understand the recommendations

☐ I needed to keep my windows open due to the hot weather

☐ I couldn’t because of costs or financial barriers (e.g., my work requires being outside, I could not afford to purchase a portable air purifier/cleaner)

☐ I did not have access to air cleaning equipment in my community (or they were sold out)

☐ There are no appropriate locations (such as available clean air refuges) in my community

☐ I did not have transportation

- ☐ I did not know about any recommendations
- ☐ I got the recommendations too late
- ☐ Other ____

Do you feel that the actions taken were helpful in reducing your exposure to wildfire smoke, which reduced negative health effects of wildfire smoke?

- ○ Yes
- ○ No
- ○ Unsure

If you were not able to take any actions, do you feel the smoke negatively affected your health?

- ○ Yes
- ○ No
- ○ Unsure

This block of questions is designed to evaluate who is responding to the survey to understand if the diversity of the province is represented by those who complete the survey and to help us determine others we need to reach.

Which of the following groups do you identify with (Please choose all that apply)?

- ☐ Living with lung disease(s)
- ☐ Living with heart disease(s)
- ☐ Older adult
- ☐ Caregiver of older adult or person(s) with chronic diseases
- ☐ Caregiver of children
- ☐ Pregnant

- ☐ None of the above
- ☐ Prefer not to say

What city do you live in or nearest to?

▼ 100 Mile House ... Prefer not to say

Supplement A

How many people, including you, currently live in your household?

- ○ 1
- ○ 2-3
- ○ 4-5
- ○ 6+
- ○ Prefer not to say

What is your age in years?

- ☐ Black (examples: African, Afro-Caribbean, African Canadian descent)
- ☐ East Asian (examples: Chinese, Korean, Japanese, Taiwanese descent)
- ☐ Southeast Asian (examples: Filipino, Vietnamese, Cambodian, Thai, Indonesian, other Southeast Asian descent)
- ☐ Indigenous (First Nations, Inuk/Inuit, Métis; examples: First Nations, Inuk/Inuit, Métis descent)
- ☐ Latino (examples: Latin American, Hispanic descent)
- ☐ Middle Eastern (examples: Arab, Persian, West Asian descent, such as, Afghan, Egyptian, Iranian, Lebanese, Turkish, Kurdish)
- ☐ South Asian (examples: South Asian descent, such as, East Indian, Pakistani, Bangladeshi, Sri Lankan, Indo-Caribbean)
- ☐ White (examples: European descent)
- ☐ Australian, New Zealander, Pacific Islands descent
- ☐ Other ____
- ☐ Prefer not to say

Which category describes you best?

- ☐ First Nations
- ☐ Inuk/Inuit
- ☐ Métis
- ☐ Two-spirit

Which best describes your current gender identity?

- ○ Man
- ○ Woman
- ○ Non-binary
- ○ Trans Man
- ○ Trans Woman
- ○ Prefer to self identify ____
- ○ Prefer not to say

How would you describe your financial situation?

- ○ Struggling to make ends meet
- ○ Enough to meet my needs
- ○ Ample disposable income
- ○ Prefer not to say

What is the highest level of education you have completed?

- ○ Less than high school
- ○ High school or equivalent
- ○ Some college or university or college diploma, degree, or certificate

- ◯ Trade/Apprenticeship
- ◯ Bachelor's degree
- ◯ Masters/PhD/Professional degree
- ◯ Prefer not to say

How did you find out about this survey?

- ◯ Email list
- ◯ Word of mouth
- ◯ BC Lung Association
- ◯ Social media
- ◯ Local air quality round table
- ◯ Other ____

As a participant in this survey, you ***may*** enter your email address to be entered into a draw to win one of seven gift cards or electronic gift cards from Tim Hortons or Walmart valued at: 1 x $100, 2 x $50, and 4 x $25. This will be drawn at the end of the survey and in no way will impact your survey results and it will be un-linked from your survey results. We will not contact you or use your email address unless you are the winner. Following the draw your email address will be deleted. As stated in the cover letter, your confidentiality will be respected and your email address will only be available to study personnel associated with Legacy for Airway Health.

- ◯ Yes (provide email address to contact you if you are a winner)
  ____
- ◯ No
