# Supplementary material for: Public Health Messaging for Wildfire Smoke: Cast a Wide Net
Source: Front Public Health. 2022 Apr 27;10:773428. doi: 10.3389/fpubh.2022.773428 (PMC9132092; doi:10.3389/fpubh.2022.773428)
Supplement: Supplementary Material B — Wildfire smoke communication survey results web app - https://ehs-bccdc.shinyapps.io/2020smoke_survey/. [file Data_Sheet_2.docx]

BC Public Engagement of wildfire smoke-related messages

- [Summary](https://ehs-bccdc.shinyapps.io/2020smoke_survey/_w_52f36d2a/#tab-6247-1)
- [Part 1](https://ehs-bccdc.shinyapps.io/2020smoke_survey/_w_52f36d2a/#tab-6247-2)
- [Part 2](https://ehs-bccdc.shinyapps.io/2020smoke_survey/_w_52f36d2a/#tab-6247-3)
- [Part 3](https://ehs-bccdc.shinyapps.io/2020smoke_survey/_w_52f36d2a/#tab-6247-4)

**Summary of survey demographics**

| **Age range** | **Percentage** | |  |
| --- | --- | --- | --- |
| Under 12 | 0.26 | |  |
| 12-17 | 0.13 | |  |
| 18-24 | 7.13 | |  |
| 25-34 | 11.62 | |  |
| 35-44 | 16.12 | |  |
| 45-54 | 15.32 | |  |
| 55-64 | 18.23 | |  |
| 65-74 | 15.59 | |  |
| 75+ | 5.28 | |  |
| Unknown | 10.30 | |  |
| **Gender identity** | | **Percentage** | |
| Man | | 30.25 | |
| Non-binary | | 0.66 | |
| Prefer to self identify | | 0.40 | |
| Trans Woman | | 0.13 | |
| Unknown | | 11.10 | |
| Woman | | 57.46 | |

| **Ethnicity** | **Percentage** |  |  |  |  |
| --- | --- | --- | --- | --- | --- |
| Non-Indigenous | 83.62 |  |  |  |  |
| Unknown | 10.70 |  |  |  |  |
| Indigenous | 5.68 |  |  |  |  |
| **Financial status** | | | **Percentage** |  |  |
| Ample disposable income | | | 26.42 |  |  |
| Enough to meet my needs | | | 50.07 |  |  |
| Struggling to make ends meet | | | 10.17 |  |  |
| Unknown | | | 13.34 |  |  |
| **Group** | | | | | **Percentage** |
| None of the above | | | | | 26.21 |
| Older adult | | | | | 25.58 |
| Living with lung disease(s) | | | | | 15.68 |
| Caregiver of children | | | | | 13.16 |
| Unknown | | | | | 7.47 |
| Caregiver of older adult or person(s) with chronic diseases | | | | | 6.95 |
| Living with heart disease(s) | | | | | 4.21 |
| Pregnant | | | | | 0.74 |

| **Urban classification** | **Percentage** |
| --- | --- |
| Metro Vancouver | 51.78 |
| Medium Population Centre | 15.19 |
| Other Urban Area | 12.15 |
| Unknown | 8.72 |
| Large Population Centre | 6.21 |
| Small Population Centre | 5.94 |

**Urban classification**

Cities that respondents are from were categorized as follows:

| **Classification** | **Population** |  |  |
| --- | --- | --- | --- |
| Metro Vancouver | Metro Vancouver |  |  |
| Other Urban Area | 80,001 + |  |  |
| Large Population Centre | 20,001 - 80,000 |  |  |
| Medium Population Centre | 5,000 - 20,000 |  |  |
| Small Population Centre | < 5,000 |  |  |
| **Ethnicity** | | | **Percentage** |
| White | | | 69.09 |
| Unknown | | | 10.70 |
| Indigenous | | | 5.68 |
| East Asian | | | 4.89 |
| Other | | | 2.38 |
| South Asian | | | 2.38 |
| Latino | | | 1.85 |
| Southeast Asian | | | 1.19 |
| Black | | | 0.79 |
| Middle Eastern | | | 0.66 |
| Australian, New Zealander, Pacific Islands descent | | | 0.40 |

| **education** | | | **percent** |
| --- | --- | --- | --- |
| Masters/PhD/Professional degree | | | 26.55 |
| Bachelor's degree | | | 27.08 |
| Trade/Apprenticeship | | | 2.51 |
| Some college or university or college diploma, degree, or certificate | | | 27.21 |
| High school or equivalent | | | 5.94 |
| Less than high school | | | 0.66 |
| Unknown | | | 10.04 |
| **How did you find out about this survey?** | **Percentage** |  |  |
| Email list | 42.14 |  |  |
| Social media | 23.91 |  |  |
| Other | 15.06 |  |  |
| No response | 8.06 |  |  |
| Word of mouth | 5.94 |  |  |
| BC Lung Association | 3.30 |  |  |
| Local air quality round table | 1.59 |  |  |

| **Other** |
| --- |
| Local networks - friends, family |
| Workplace |
| Reach BC |
| VCH website |
| Community newsletters |
